# Surveying Therapists on Seating Approaches for Patients with Muscular Dystrophy in Japan

**DOI:** 10.3390/healthcare9060631

**Published:** 2021-05-25

**Authors:** Hitomi Fujita, Atsushi Tsukada, Tomoko Ohura

**Affiliations:** 1 Department of Rehabilitation, Faculty of Health Sciences, Nihon Fukushi University, Aichi 475-0012, Japan; 2 Department of Mechanical Engineering, Faculty of Science and Technology, Meijo University, Nagoya 468-8502, Japan; tsukada@meijo-u.ac.jp; 3 Division of Occupational Therapy, Department of Rehabilitation, Faculty of Health Sciences, Naragakuen University, Nara 631-8524, Japan; oh-ura@umin.ac.jp

**Keywords:** muscular dystrophy, occupational therapists, physiotherapists, seating, survey, wheelchair

## Abstract

Patients with muscular dystrophy (MD) need fitted wheelchairs. This study aimed to ascertain physiotherapists and occupational therapists’ opinions about the current wheelchair seating process for patients with MD in Japan. We identified 266 academic papers published between August 2014 and July 2019 with the keywords “muscular dystrophy” and either “physiotherapy” or “occupational therapy.” We then sent survey requests to 140 physiotherapists and occupational therapists (who were among the authors of the aforementioned papers), of whom 41 agreed to partake in this study. We found that the time required for each seating was 30–60 min for three types of MD, and the most commonly reported time to trial fitting was 1–3 months. In addition, health insurance reimbursements for seating were considered part of disease-specific rehabilitation in most cases, and most therapists were more or less satisfied with the current seating procedure. Physiotherapists had the highest degree of reflection of their views (wishes) regarding seating, followed by MDs and their families. In Japan, seating has been regarded as a medical practice since 2017. In the future, we would like to investigate the seating concept for individual therapists in detail.

## 1. Introduction

Muscular dystrophy (MD) is an intractable disease characterized by the onset of progressive muscle weakness and atrophy in childhood, for which no curative treatment has yet been established [1]. Although the latest drug treatments slow disease progression [2], attention is being placed on improving patients’ quality of life (QOL) and efforts are being made to focus on patients’ transition to adulthood [3,4]. An area of concern regarding QOL is the wheelchair, which is an important assistive device that enables patients to participate socially at school age and older. Most Japanese patients with MD are provided with postural support devices with wheelchairs and other mobility devices before the development of spinal deformity to prevent the progression of spinal deformity and improve their activities of daily life [5]. The importance of acquiring the habit of maintaining a good seated posture from around the start of elementary school is emphasized in the *Treatment and Care Manual for Spinal Deformity in Muscular Dystrophy* [6]. For the provision of a wheelchair (seating) that is appropriate to a patient’s physical function, the choice of device, method of use, and specifications must be considered by doctors and professionals in multiple fields [7], and the absence of comprehensive support may lead to the development of functional issues [8].

One characteristic of seating for patients with pediatric neurological diseases is that the capabilities that they can utilize vary depending on the location of both the lower limbs, neck, and trunk. As most patients start using a wheelchair during childhood or adolescence, when both mental and physical functions gradually improve, it is essential to respond rapidly to changes over time. Patients with MD generally switch from a manual wheelchair to a power-assisted or motorized wheelchair as their motor disability progresses [1]. Previous studies indicate that early intervention is recommended before symptom progression [9] and consideration for trunk function is required [10]; accordingly, severe secondary damage may occur if adjustments are not made in accordance with functional disability [11]. In addition, seating is recommended as an intervention to prevent or improve the limited range of motion [12]. However, because MD is a rare disorder and patients have a short life expectancy, it is difficult to investigate its long-term prognosis using interventional studies [13]. Wheelchairs are used after patients with MD lose their ability to walk; MD patients live on wheelchair support for more than half of their lives and thus wheelchairs have a major effect on their social participation [14]. Therefore, further studies are needed to improve MD patients’ QOL as their life expectancy increases [3,4].

Wheelchairs and electric beds are considered to be medical devices in the United States and Europe and are registered as medical devices in China. However, in Japan, wheelchairs are regarded as non-therapeutic prostheses and assistive devices. Welfare equipment methods were drawn into law in anticipation of the aging of the population in 1993 [15], and Long-Term Care Insurance Law was enacted in 2000 [16]. As a result, the welfare equipment loan system began, and seating focused on elderly people in need of long-term care. Furthermore, seating became a target for calculating medical fees in 2017 [17], and seating has a medical aspect in Japan as well.

Although some studies have described seating procedures, few studies have addressed the provision process, and the situation of intervention remains unclear. In the clinical setting, patients are also consulted, and it is presumed that seating procedures will greatly influence patient’s QOL [18]. Wheelchairs are an indispensable means of transportation for patients with MD, especially for social activities, and seating contributes to improving their QOL. Since seating in Japan has only recently become a medical practice [17], we conducted a survey among expert therapists as the first step to consider what decision-making process would be desirable.

## 2. Materials and Methods

### 2.1. Inclusion Criteria for Therapists

The subjects were therapists who practiced seating for patients with MD in Japan. The number of people who are professionally involved in seating in Japan is limited, and no comprehensive list of such clinicians exists. Thus, to determine the inclusion criteria, we identified 266 academic papers with the keywords “muscular dystrophy” and either “physiotherapy” or “occupational therapy” published by physiotherapists (PTs) or occupational therapists (OTs) over a 5-year period: between August 2014 and July 2019. The Ichushi-web (Japanese medical literature) was used as a database. We then sent survey requests to a total of 140 PTs and OTs who were among the authors of these papers. Of the 140 PTs and OTs, 41 agreed to take part in the study, 34 declined, 19 had moved, and 46 did not reply. In all cases, the reason for declining to take part in the study was “I am not involved in seating patients with MD.” Questionnaires were returned by the 41 therapists who agreed to participate.

### 2.2. Study Process

#### 2.2.1. Preliminary Survey

Before the main survey, we carried out a pilot survey of clinics that had agreed to cooperate with the study to solicit their opinions on the matters covered by the survey. We asked the PTs of the cooperating facilities to answer the draft questionnaire: (1) therapists’ information, (2) questions (asked separately) about different types of MD, and (3) questions on the procedures involved in seating patients with MD; responses were confirmed by a direct interview. A misunderstood question was corrected, and another question—(4) general questions on seating—was added to the survey to incorporate therapists’ educational and empirical background. All surveys were conducted in Japanese.

#### 2.2.2. Consent, Mail-Out, and Return of the Survey

All the therapists provided written consent for participation. The therapists were mailed a questionnaire to complete and return.

### 2.3. Ethical Considerations

Ethical considerations concerning the respondents and the therapists who partook in this study were approved by the Ethics Committee on Research with Human Subjects at Nihon Fukushi University (approval number, 18–48). All the respondents were provided with a written explanation of the purpose, significance, and ethical considerations of the study, and they gave their consent to participate in this study.

### 2.4. Survey Content

The questionnaire covered the items listed in Table 1. Eight questions were used to obtain information about each therapist (including type of institution of employment, provision of seating by institution, sex, age, type of profession licensing, the length of time since a license was obtained, the number of patients seated, and the length of time in seating experience), four questions regarded the different types of MD—Duchenne MD, Fukuyama MD, and other MD (including seating for patients with different types of MD, the length of time required for each seating, the length of time to trial fitting, and length of time from trial fitting to handover), four questions regarded the procedures involved in seating patients with MD (including the method of calculating health insurance reimbursements, whether views (or wishes) were reflected [measured using a 10-point Likert scale], whether a particular approach to seating was used [if respondents selected Yes, they were asked for the name and viewpoints of the approach], the level of satisfaction with seating, and any issues experienced with seating), and five general seating questions were asked (respondents could choose single or multiple answers). In several questions, if options were not applicable, respondents had the option of selecting “Other”.

### 2.5. Method of Analysis

Basic statistics for all the survey items were performed and R ver. 1.42 was used for statistical analysis. Data are expressed as the mean and standard deviation.

## 3. Results

Before conducting the survey, we reviewed 170 papers published in Japan; there were only five case studies on postural support by seating. Our questionnaire was sent to 41 therapists and answered by 38 (a 93% response rate).

### 3.1. Therapists’ Information

Details about therapists’ (respondents) information are summarized in Table 2.

Thirty respondents indicated that they were employed by a hospital, two by a clinic, one at a center for children with disabilities, and one at a home-visiting service. Other therapists responded that they worked at both hospital and home-visiting services. The majority (82%) of therapists responded that the institutions where they were employed provided seating when required by the situation, and six responded that seating was only provided on specific days or times.

Based on the profession, 28 PTs and 10 OTs responded. There were 30 men and the mean age was 40.1 ± 8.6 years. The mean number of years since their PT or OT license was obtained was 16.4 ± 9.1 years. The mean length of experience in seating patients with MD was 11.6 ± 8.6 years, which was approximately 5 years less than the time elapsed since their licenses were obtained.

More than half of the respondents had provided seating for at least 10 patients, who were not limited to patients with MD.

### 3.2. Seating for Patients with Different Types of MD

The responses to the questions about seating for different types of MD are summarized in Table 3. “Other” refers to MDs of types other than Duchenne MD and Fukuyama MD.

Therapists were asked whether inpatients and outpatients were treated differently. For each type of MD, some institutions treated only inpatients, but a few institutions only treated outpatients; however, for all three types of MD, most therapists responded that they treated both inpatients and outpatients (Duchenne MD, 14 respondents; Fukuyama MD, 9 respondents; other MDs, 11 respondents). Most therapists responded that the length of time required for each seating was 30–60 min for all three types of MD (Duchenne MD, 20 respondents; Fukuyama MD, 10 respondents; other MD, 19 respondents). However, some therapists responded that >60 min were required for seating. The most commonly reported length of time to trial fitting was 1–3 months (Duchenne MD, 15; Fukuyama MD, 7; other MD, 13), and the most commonly reported that the length of time from trial fitting to handover was also 1–3 months.

### 3.3. Questions on Procedures Involved in Seating patients with MD

The responses to questions about procedures involved in seating patients with MD are summarized in Table 4.

Health insurance reimbursements for seating were considered a part of disease-specific rehabilitation in most cases. In a few cases, however, seating was not included in the reimbursement calculations. Respondents were asked to grade the extent to which the opinions (or wishes) of the people directly concerned were reflected in seating, using a 0–10-point scale. Over half of the therapists responded that a particular approach toward seating was adopted. Finally, for the question about whether they were satisfied with the current seating procedure, most therapists responded that they were more or less satisfied, followed by the somewhat dissatisfied response.

### 3.4. General Questions on Seating

General questions on seating were not limited to patients with MD (Table 5).

Half of the therapists had not attended a workshop providing seating-related knowledge or skills in the past 3 years. The majority of therapists (25), however, responded that they would attend a workshop providing seating-related knowledge or skills in the future. The respondents were divided into those who were continuing to attend seating workshops and other educational events and those who neither had attended a workshop or educational event during the past 3 years nor would do so in the future. Most of the institutions where respondents were employed were willing to allow therapists to attend workshops and other educational opportunities.

Multiple responses were possible to the question concerning how therapists obtain information about seating. The answers showed that PTs and OTs obtained information mostly from experienced or knowledgeable therapists or acquaintances, followed by workshops and seminars, specialist publications, the internet, and other sources, including wheelchair vendors. In terms of their own level of understanding of seating, more than half of the PTs and OTs responded that they mostly understood it. This level of understanding was based on the response, “I have the knowledge to deal with requests for advice from people directly concerned.”

## 4. Discussion

The average age of the 28 PTs and 10 OTs who responded to our survey was higher than the mean age of all PTs and OTs in Japan (34.0 years for PTs and 34.6 years for OTs) [19,20], the mean number of years since licensing was 16.4 ± 9.1 years, and many PTs and OTs had provided seating for ≥10 patients, indicating that these PTs or OTs had been involved in seating patients with MD for many years.

Most therapists responded to the question concerning the provision of seating by the institution where they were employed by saying that “seating is provided when required depending on the situation,” indicating that institutions were flexible for seating patients with MD depending on the timing and circumstances of requests for advice from patients with MD. Regarding whether their institutions provided seating to inpatients or outpatients, the results suggested that seating is mostly provided as an outpatient service to patients in stable conditions that do not require inpatient treatment and that may be admitted to the hospital for their seating to be adjusted when their condition changes. According to Richardson et al. [9], early intervention and regular checkups are essential to prevent preemptive problems; Richardson et al. also state that intervention (such as postural support) is required at the early stage when the individual is still able to stand or can walk a little before deformity develops or before an individual can accept their present posture or postural changes. A few respondents stated that the length of time required to seat a patient was <30 min, the majority responded that 30–60 min was required for seating, and 10 respondents answered that >60 min was required for patients with Duchenne MD. These findings suggest that the time required for seating varies depending on the nature of the task, and on average, takes around 1 h. Although a number of guidelines [12,21] on seating methods have been published, the actual time required to seat a patient remains unknown. The time required to seat a patient includes the time required for a detailed assessment of postural changes over time and the patient’s physical reaction time to adjustments made to the device.

The questions about procedures involved in seating included one question on the calculation of health insurance reimbursements. Some therapists answered that reimbursements were calculated for seating alone, and others answered that reimbursements were calculated as part of disease-specific reimbursements or that seating was not included in reimbursement calculations. A study by Costigan et al. [22] demonstrated that individual reimbursements are not given under the direct primary care system, which is evident in from some of the responses obtained in our study. In addition, given that most of the respondents stated that at least 30 min was required for each seating, the actual time required might not have been reflected in the calculations.

We also asked about the level to which the opinions (or wishes) of various people directly concerned in seating were reflected during the procedure. PTs were the most likely group to have their views reflected, followed by the patients themselves and their family members. Because seating for patients with MD involves a wide range of medical aspects, from postural support in everyday life to considerations of risk, the involvement of PTs has a major effect on seating. Where powered wheelchairs were concerned, many respondents noted that OTs were mainly involved in the use of the controller to operate the chair. This finding indicates that there is a division of roles between these professionals [23]. Many therapists stated that, although patients have their own opinions about how convenient devices should be used, as well as pain and other physical effects, it may not be possible to fulfill all the patient’s requests due to problems with physical function or issues with administrative procedures; this factor reduces the degree to which patients’ views are reflected. In addition, intellectual factors may also make it difficult to elicit specific wishes, indicating that the decision may be left to the discretion of the patient’s family members and people concerned directly [24].

Family members often look for assistance, and from their perspective, a wheelchair for postural support may not be the most effective tool for enabling caregivers to provide assistance [25]. Therefore, it is important to fully explain all procedures to family members and share their understanding, as the goal of seating may vary greatly depending on the type of lifestyle envisaged by the patient and their family and on their value [26].

Of the 20 respondents who stated that they use a specific approach for seating, nine mentioned the Active Balance Seating [27] and one mentioned the Caput-Axis-Skeleton-Proportion-Enjoy-Relax approach [28]. Other comments to this question included descriptions of the specific nature of the intervention, and similar comments were made by therapists who stated that they did not use a specific approach. This finding indicates that therapists carried out seating interventions based on a concept. Although therapists’ level of satisfaction with seating was generally high, some did show a low level. Further studies are required to identify which factors are associated with satisfaction.

Five questions covered seating in general. The responses showed that the levels of previous and intended workshop attendance and the willingness of institutions to allow workshop attendance were all relatively good. Knowledge and information about seating were obtained using multiple methods. Workshops and seminars on seating are now available via a range of different methods. Although these approaches are likely to be helpful when responding immediately in everyday clinical practice, they also include information that has not been fully scientifically validated. Indeed, as mentioned above, only a small number of scientific papers on the process of seating for postural support have been published in Japan.

The limitation of this study is that the number of people surveyed was small. The 38 people who became collaborators in the survey constituted only a small part of the number of PTs and OTs in Japan; hence, the results are limited in their representation. However, those who did not participate in the survey did not include those who were involved in the seating of patients with MD. Moreover, the recovery rate was high and the seating of patients with MD was concentrated to some facilities. Our future plans involve conducting a qualitative survey to extract deeper issues within a limited number of subjects.

## 5. Conclusions

In this study, we conducted a survey of physiotherapists and occupational therapists involved in the seating of patients with MD in Japan. As a result of the survey, many therapists were found to have clinical experience and to calculate a part of the time required to seating as a medical fee. It was also revealed that the opinions of PTs, patients with MD, and families were mainly incorporated into the seating, and that while many therapists were satisfied with the seating, some were dissatisfied. In the future, we will focus on the problems felt by individual therapists and examine what decision-making processes are desirable for seating patients with MD.

## Figures and Tables

**Table 1:** Survey Content.

Area	Question
Therapists’ information	Type of institution where employed
	Provision of seating by institution where employed
	Sex
	Age
	Profession (physiotherapist or occupational therapist)
	Number of years since licensing was obtained
	Length of experience in seating patients with MD
	Number of patients with MD seated
Questions (asked separately) for different types of MD *	Type of patients for whom seating is provided (inpatients/outpatients/both/none)
	Length of time required for each seating
	Length of time until first trial fitting
	Length of time from first trial fitting to initial handover
Questions on procedures involved in seating patients with MD	Method of calculating health insurance reimbursements
	Views reflected in seating and individuals consulted
	Use of a particular approach to seating
	Specific approach (name and viewpoint)
	Current level of satisfaction with seating
	Any issues experienced with seating
General questions on seating	Participation in a workshop in the past 3 years
	Future participation in a workshop providing seating-related knowledge or skills
	Willingness of institution where the respondent is employed to allow participation in workshops
	Methods of obtaining knowledge of and information about seating
	Own level of understanding of seating

* These included (i) Duchenne MD, (ii) Fukuyama MD, and (iii) other MD.

**Table 2:** Characteristics of respondents (*n* = 38).

Question	Choice	*n*	(%)
Type of institution where the respondent is employed	Hospital	30	(78.9)
	Clinic	2	(5.3)
	Center for children with disabilities	1	(2.6)
	Home-visit rehabilitation	1	(2.6)
	Child welfare facility	0	(0.0)
	Other	3	(7.9)
Provision of seating by institution	Specific days or times	6	(15.8)
	When required by the situation	31	(81.6)
	Other	1	(2.6)
Sex	Male	30	(78.9)
	Female	8	(21.1)
Age (years)		40.1 ± 8.6 *	
Profession licensing type	Physiotherapist	28	(73.7)
	Occupational therapist	10	(26.3)
Number of years since license was obtained(years)		16.4 ± 9.1 *	
Length of experience in seating (years)		11.6 ± 8.6 *	
Number of patients seated	<5	11	(28.9)
	5–10	6	(15.8)
	>10	19	(50.0)

* mean ± SD.

**Table 3:** Seating for patients with different types of MD (*n* = 38).

		(i) Duchenne		(ii) Fukuyama		(iii) Other	
Question	Choice	*n*	(%)	*n*	(%)	*n*	(%)
Inpatient/outpatient	(1) Inpatient	9	(23.7)	6	(15.8)	6	(15.8)
	(2) Outpatient	10	(26.3)	4	(10.5)	10	(26.3)
	(3) Both	14	(36.8)	9	(23.7)	11	(28.9)
	(4) None	5	(13.2)	19	(50.0)	11	(28.9)
Length of time required for each seating	(1) <30 min	2	(6.3)	1	(5.3)	3	(10.7)
	(2) 30–60 min	20	(62.5)	10	(52.6)	19	(67.9)
	(3) >60 min	10	(31.3)	8	(42.1)	6	(21.4)
Length of time to trial fitting	(1) <1 month	11	(33.3)	7	(35.0)	8	(28.6)
	(2) 1–3 months	15	(45.5)	7	(35.0)	13	(46.4)
	(3) 3–6 months	7	(21.2)	6	(30.0)	7	(25.0)
	(4) >6 months	0	(0.0)	0	(0.0)	0	(0.0)
Length of time from trial fitting to handover	(1) <1 month	3	(9.1)	3	(15.0)	3	(10.3)
	(2) 1–3 months	14	(42.4)	8	(40.0)	12	(41.4)
	(3) 3–6 months	12	(36.4)	6	(30.0)	12	(41.4)
	(4) >6 months	4	(12.1)	3	(15.0)	2	(6.9)

**Table 4:** Questions about procedures involved in seating (*n* = 38).

Question	Choice	*n*	(%)
Method of calculating health insurance reimbursements	(1) Calculated for seating only	6	(15.8)
	(2) Calculated as part of disease-specific rehabilitation	26	(68.4)
	(3) Not calculated	6	(15.8)
Level to which views (wishes) are reflected *	(1) Patient	7.5 ± 1.9	
	(2) Family members	7.3 ± 1.6	
	(3) Welfare service providers other than family members	5.3 ± 2.8	
	(4) Doctors	4.7 ± 3	
	(5) Physiotherapists	7.6 ± 1.8	
	(6) Occupational therapists	6.6 ± 2.2	
	(7) Orthotists	5.4 ± 2.5	
	(8) Wheelchair producers	6.5 ± 1.9	
	(9) Teachers at the patient’s school	5.2 ± 2.3	
	(10) Other **	5.7 ± 3.1	
Use of particular approach to seating	(1) Yes	20	(52.6)
	(2) No	18	(47.4)
Satisfaction with seating	(1) Very satisfied	1	(2.6)
	(2) More or less satisfied	25	(65.8)
	(3) Somewhat dissatisfied	11	(28.9)
	(4) Very dissatisfied	1	(2.6)

* mean ± SD. ** Rehabilitation doctors, staff of centers for disabled children, and ward staff were mentioned.

**Table 5:** General questions about seating (*n* = 38).

Question	Choice	*n*	(%)
Attendance at a workshop providing seating-related knowledge or skills in the past 3 years	(1) Yes	17	(44.7)
	(2) No	19	(50.0)
	(3) Other	2	(5.3)
Future workshop attendance	(1) Yes	25	(65.8)
	(2) No	9	(23.7)
	(3) Other	4	(10.5)
Willingness of institution where the respondent is employed to allow workshop attendance	(1) Yes	36	(94.7)
	(2) No	2	(5.3)
	(3) Other	0	(0.0)
Method of obtaining knowledge and information about seating (multiple responses allowed)	(1) Therapists and acquaintances	33	(86.8)
	(2) Internet	20	(52.6)
	(3) Specialist publication	21	(55.3)
	(4) Workshops and seminars	25	(65.8)
	(5) Unable to locate such information	0	(0.0)
	(6) No means of obtaining such information	1	(2.6)
	(7) No need for such knowledge	0	(0.0)
	(8) Other	4	(10.5)
Level of understanding of seating	(1) Fully understand	2	(5.3)
	(2) Mostly understand	24	(63.2)
	(3) Don’t understand very well	11	(28.9)
	(4) Don’t understand at all	0	(0.0)
	(5) Other	1	(2.6)

## Data Availability

Data generated and analyzed during this study are included in this article. Additional data are available from the corresponding author on request.
